# Heparanase expression upregulates platelet adhesion activity and thrombogenicity

**DOI:** 10.18632/oncotarget.8960

**Published:** 2016-04-23

**Authors:** Hao Cui, Ying-xia Tan, Cecilia Österholm, Xiao Zhang, Ulf Hedin, Israel Vlodavsky, Jin-Ping Li

**Affiliations:** ^1^ Department of Medical Biochemistry and Microbiology, SciLifeLab Uppsala, The Biomedical Center, University of Uppsala, Husargatan, Uppsala, Sweden; ^2^ Department of Tissue Engineering, Beijing Institute of Transfusion Medicine, Beijing, China; ^3^ Department of Molecular Medicine and Surgery, Karolinska Institutet, Stockholm, Sweden; ^4^ Cell Therapy Institute, Nova Southeastern University, Fort Lauderdale, FL, USA; ^5^ Department of Neuroscience and Pharmacology, University of Uppsala, Husargatan, Uppsala, Sweden; ^6^ Cancer and Vascular Biology Research Center Rappaport, Faculty of Medicine, Technion, Haifa, Israel

**Keywords:** heparanase, heparan sulfate, platelet, platelet adhesion, thrombosis

## Abstract

Heparanase is an endo-glucuronidase that specifically cleaves heparan sulfate (HS) and heparin polysaccharides. The enzyme is expressed at low levels in normal tissues, but is often upregulated under pathological conditions such as cancer and inflammation. Normal human platelets express exceptionally high levels of heparanase, but the functional consequences of this feature remain unknown. We investigated functional roles of heparanase by comparing the properties of platelets expressing high (Hpa-tg) or low (Ctr) levels of heparanase. Upon activation, Hpa-tg platelets exhibited a much stronger adhesion activity as compared to Ctr platelets, likely contributing to a higher thrombotic activity in a carotid thrombosis model. Furthermore, we found concomitant upregulated expression of both heparanase and CD62P (P-selectin) upon activation of mouse and human platelets. As platelets play important roles in tumor metastasis, these findings indicate contribution of the platelet heparanase to hyper-thrombotic conditions often seen in patients with metastatic cancer.

## INTRODUCTION

Platelets are primary mediators of blood coagulation. Upon blood vessel injury, platelets are activated and aggregated at the site of injury[1]. Apart from forming a primary hemostatic plug, activated platelets degranulate and release a spectrum of active molecules from the granules. Dense granules release active ligands including ADP/ATP to propagate platelet aggregation. Alpha-granules release, in addition to pro-coagulant proteins, also anti-coagulation molecules such as antithrombin and fibrinolytic proteins, e.g. plasminogen. Emerging information points to additional biological roles of platelets, attributed to active molecules secreted upon their activation and to the release of microparticles that mediate cellular crosstalk[2]. For example, platelets directly and indirectly promote tumor growth and protect metastasizing cells to escape T-cell-mediated immunity and natural killer cell surveillance[3, 4]

Heparanase is an endo-glucuronidase that specifically cleaves heparan sulfate (HS) and heparin polysaccharide chains. In humans heparanase enzymatic activity was initially detected in platelets [5], most likely owing to the relatively high expression level of the enzyme in platelets compared to essentially non-detectable amounts in the majority of other normal tissues. Notably, heparanase is elevated in a number of human pathological tissues, e.g. cancer[6] and its activity is implicated in neovascularization, inflammation, and autoimmunity[7, 8]. To this date, the biological functions of heparanase are mainly attributed to its enzymatic activity. Heparanase modulates the extracellular matrix (ECM) and basement membrane structures through degradation of heparan sulfate proteoglycans (HSPGs), which leads to release of ECM-bound active molecules and subsequent cell proliferation, cell migration and signal transduction[8]. Recent reports, on the other hand, ascribe the pro-coagulant function of heparanase to non-enzymatic activities of the protein[9]. Taking into account that the enzyme is abundantly expressed in human platelets, it is of importance to elucidate its functional roles in coagulation and thrombosis.

Overexpression of heparanase in transgenic mice results in degradation of HS accompanied by diverse phenotypes that are plausibly related to alterations in HS structure[10–12]. Specifically, a recent study demonstrated increased thrombosis in heparanase-overexpressing mice in response to vascular injury and stent-induced flow disturbance[13], further supporting a role of heparanase in hemostasis.

In this work, we aimed at studying the pathophysiological implications of heparanase in platelet activity. Results obtained from *in vivo* and *in vitro* experiments using wild type and heparanase overexpressing C57BL mice indicate that increased heparanase expression level is associated with platelet adhesion and spreading. These effects of heparanase have important clinical implications, e.g., in patients with hematological and neoplastic disorders.

## RESULTS

### Characterization of platelets

**Figure 1:**
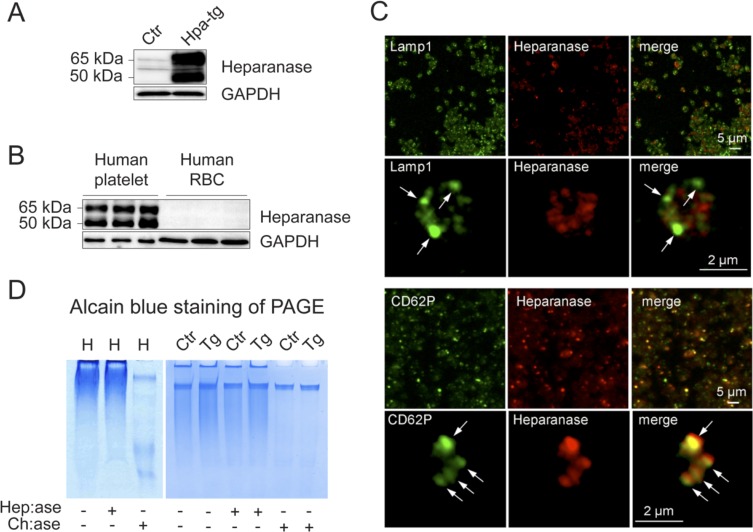
Expression of heparanase and GAGs in human and mouse platelets **A.**, **B.** Protein extracts (40 μg) from washed platelets and red blood cells (RBC) were analyzed by Western blotting using anti-heparanase antibody (#1453). **A.** Ctr mouse platelets express minute amounts of latent (65kDa) heparanase, while Hpa-tg platelets express both latent (65kDa) and active (50kDa) forms. **B.** Analysis of blood cells from 3 healthy individuals shows that both the latent (65kDa) and active (50kDa) forms of heparanase are abundantly expressed in platelets, while barely detectable in RBC. **C.** Microscopic analysis of Hpa-tg platelets adhered on collagen (100 μg/ml) coated coverslips shows co-localization of heparanase with CD62P in alpha granules. The cells were immunostained with Lamp1 (marker for dense granules and lysosome; arrows in the 2nd row of panels) and CD62P (marker for alpha granules; arrows in the bottom panels). **D.** Total proteoglycans isolated from human (H; left panel) and mouse (Ctr: control; Tg: Hpa-tg; right panel) platelets were treated with bacterial heparinases I, III (Hep:ase, degrading HS and heparin) or chondroitinase ABC (Ch:ase, degrading chondroitin sulfate) followed by separation on PAGE and Alcian blue staining. Proteoglycans appear as smear in the PAGE due to heterogeneity of the GAG chains that were resistant to heparinases but were degraded by chondroitinase ABC.

Overexpression of both the latent 65 kDa and active 50 kDa heparanase in platelets from mice overexpressing heparanase (Hpa-tg) is confirmed by Western blot analysis (Figure 1A). Examination of platelets and red blood cells (RBC) from three individuals (healthy volunteers) demonstrates that human platelets express high levels of heparanase as compared to undetectable levels in human RBC (Figure 1B). Immunostaining and fluorescence microscopy analysis of platelets from Hpa-tg mouse showed co-localization of heparanase with CD62P (P-selectin) in alpha-granules (Figure 1C). Proteoglycans purified from human (left panel) and mouse (right panel) platelets were analyzed by electrophoresis (Figure 1D). Alcian blue staining following PAGE separation revealed that glycosaminoglycans (GAG) from platelets were resistant to bacterial heparinases, but susceptible to chondroitinase, indicating that both human and mouse platelets express chondroitin sulfate (CS), but are devoid of HS. Transgenic overexpression of heparanase did not affect expression of CS in mouse platelets, as GAG samples from Hpa-tg and Ctr platelets exhibited a similar migration pattern on PAGE, with or without the enzymatic treatments. This was also confirmed by Q-PCR analysis of serglycin (*Srgn*) mRNA expression (Figure S1), which is the core protein of CS proteoglycans.

### Effect of heparanase on platelet adhesion

**Figure 2:**
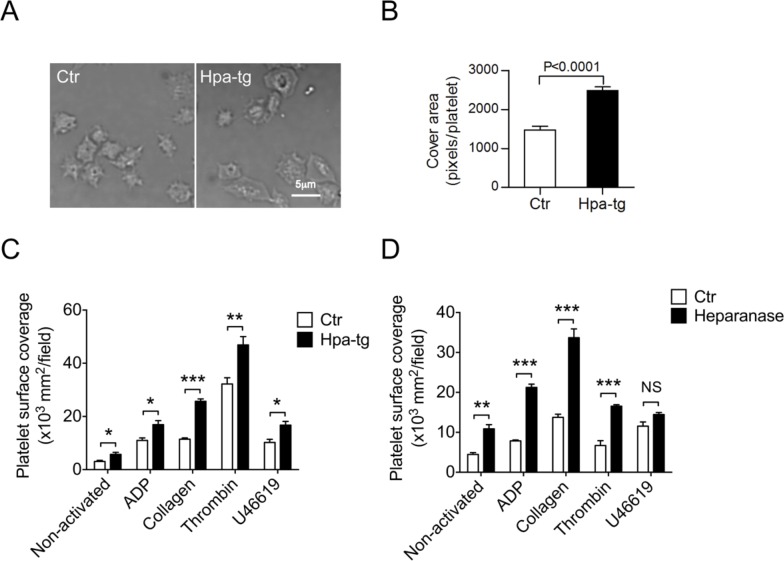
Higher adhesion and spreading activity of Hpa-tg platelets **A.** Platelets from Hpa-tg and Ctr mice were incubated on fibrinogen coated coverslips for 30 min. Images of adhering platelets were taken by differential interference contrast (DIC) microscopy with a Plan Apo VC 60×/1.40 oil immersion lens. **B.** Pixels of each platelet attached to the slide were quantified with Image J software. A total of 25 platelets from each group of animals were determined for the statistical analysis using GraphPad Prism 5.0. **C.** Equal number of platelets diluted in Tyrode's buffer was added onto monolayer of HUVEC in presence of the stimulators indicated. After incubation for 30 min at 37°C, the non-adhered platelets were rinsed away with PBS, and the adhered cells were visualized by staining with anti-CD61 and quantified by Image J software. **D.** HUVEC monolayer was first incubated with recombinant heparanase (2μg/200 μl) followed by incubation with fluorescent platelets isolated from GFP-expressing C57BL mice in the presence of activators as indicated. The data are mean ± SE of 4 mice in each group.

Correlation of heparanase overexpression with platelet activity was examined by analysis of platelet adhesion and spreading by comparing platelets from Hpa-tg *vs*.Ctr mice. First, we examined adhesion of platelets to fibrinogen-coated coverslips. Upon activation with ADP, the platelets were allowed to adhere and spread for 30 min. Analysis by differential interference contrast (DIC) microscopy revealed stronger adhesion capacity of Hpa-tg *vs*. Ctr platelets, as evaluated by their morphology (Figure 2A). The stronger spreading of Hpa-tg platelets was confirmed by quantification of pixels per platelet on the slide (Figure 2B). Next, we assessed adhesion of platelets on cultured HUVEC cells upon activation (Figure S2). Equal numbers of platelets purified from Hpa-tg and Ctr mice and diluted in Tyrode's buffer were co-cultured on confluent monolayers of HUVEC in the presence of the stimulators (ADP, collagen, thrombin and U46619) for 30 min. After washing off the non-adhered platelets, the adhered platelets were stained with anti-CD61 antibodies and visualized under a fluorescence microscope. Quantification of CD61 positive areas on the HUVEC cell surface revealed a significantly higher adhesion of Hpa-tg platelets upon activation with the stimulators (Figure 2C), suggesting that heparanase expression increased the adhesion of platelets to endothelial cell layers. As activation of platelets leads to release of granule contents including heparanase, we assumed that the stronger adhesion ability of Hpa-tg platelets maybe due, at least partly, to degradation of HS expressed on the cell surface of HUVEC. To verify this, we pre-treated the cultured HUVEC with heparanase before co-culturing with activated platelets isolated from Ctr mice. The results clearly show that pre-treatment of HUVEC significantly increased the adhesion of wildtype platelets (Figure 2D).

### Activation of platelets stimulates expression of heparanase and CD62P

**Figure 3:**
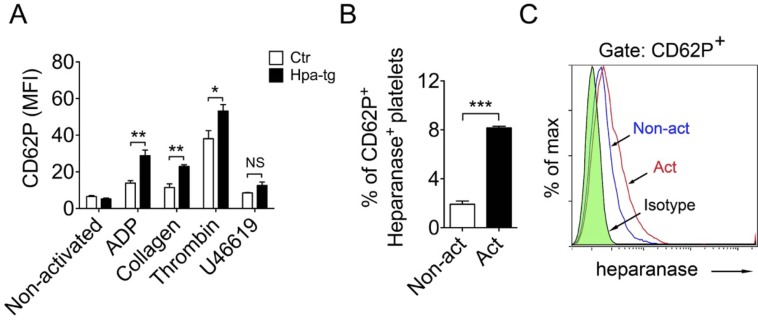
Association of heparanase and CD62P expression in mouse and human platelets upon activation **A.** Blood cells were collected from Hpa-tg and Ctr mice and activated by addition of the stimulators as indicated. After staining with antibodies, the cells were analyzed by FACS. Total of 10,000 platelets (confirmed by monoclonal antibody CD61-PE staining) were counted. Data are mean ± SE of 4 mice. **B.**, **C.** Similar FACS analysis of human blood cells from 3 healthy individuals. CD62P^+^ *vs*. heparanase^+^ platelets are expressed as percentage of total platelets. Total of 10,000 platelets were counted.

We have observed co-localization of heparanase and CD62P in alpha-granules of Hpa-tg platelets (Figure 1C). Further, microscopy analysis confirmed undetectable expression of CD62P and heparanase in non-activated Ctr platelets (Figure S3; upper panels), whereas ADP activated Ctr platelets exhibited increased expression of both CD62P and heparanase (Figure S3; middle upper panels). In contrast, substantial expression of both CD62P and heparanase was observed in non-activated Hpa-tg platelets (Figure S3; middle lower panels), and activated Hpa-tg platelets showed an intensely increased expression of both proteins (Figure S3; lower panels). To verify the immunocytostaining results, we examined CD62P expression by FACS analysis. The Hpa-tg platelets showed significantly higher expression of CD62P upon activation by ADP, collagen and thrombin in comparison to Ctr platelets. However stimulation with U46619 yielded only marginally increased expression of CD62P in both Hpa-tg and Ctr platelets (Figure 3A). Further, FACS analysis revealed a significant increase of CD62P and heparanase in activated human platelets (Figure 3B, 3C), confirming that activation trigged expression of both CD62P and heparanase in normal human platelets.

### Suppressed expression of integrin β1 and β3 in platelets overexpressing heparanase

**Figure 4:**
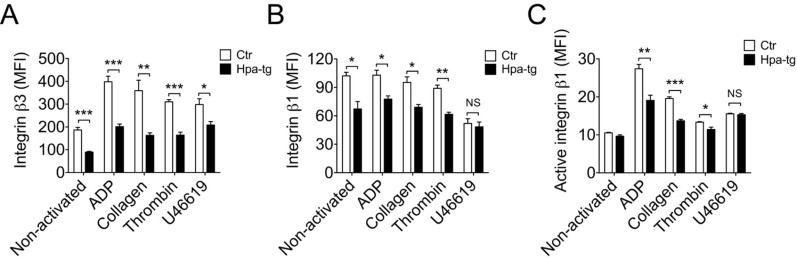
Reduced expression of integrin β3 and β1 in Hpa-tg platelets Blood cells from Hpa-tg and Ctr mice were collected and incubated with the stimulators as indicated. After staining with antibodies against integrin β3 **A.**, β1 **B.** and active integrin β1**C.**, the cells were analyzed by FACS and the signals (MFI) were quantified. Total of 10,000 platelets were counted and data are mean ± SE of 4 mice.

Considering that integrins constitute major receptor family in platelets, it can be assumed that the higher activity of the Hpa-tg platelets is associated with integrin activity. To find out expression of integrins, platelets were activated with the 4 different stimulators and analyzed by FACS following staining with the corresponding antibodies. Unexpectedly, we found lower basal level of integrin β3 in Hpa-tg platelets in comparison to Ctr (Figure 4A). Incubation with the activators increased expression of integrin β3 in both Hpa-tg and Ctr platelets; however, the level in Hpa-tg was still significantly lower than that in Ctr platelets. Similarly, integrin β1 expression level was significantly lower in Hpa-tg platelet than in Ctr (Figure 4B). Stimulation with ADP, collagen and thrombin did not affect its expression, in agreement with earlier observations[14], though U46619 stimulation led to reduced expression of integrin β1. However, the level of active integrin β1 appears comparable in non-activated Hpa-tg and Ctr platelets (Figure 4C). Active integrin β1 expression was increased upon activation in both Hpa-tg and Ctr platelets; again Hpa-tg exhibited a significantly lower level than Ctr platelets. These data exclude a direct correlation of integrin activity with the increased Hpa-tg platelet adhesion.

### Increased carotid artery thrombosis in mice overexpressing heparanase

**Figure 5:**
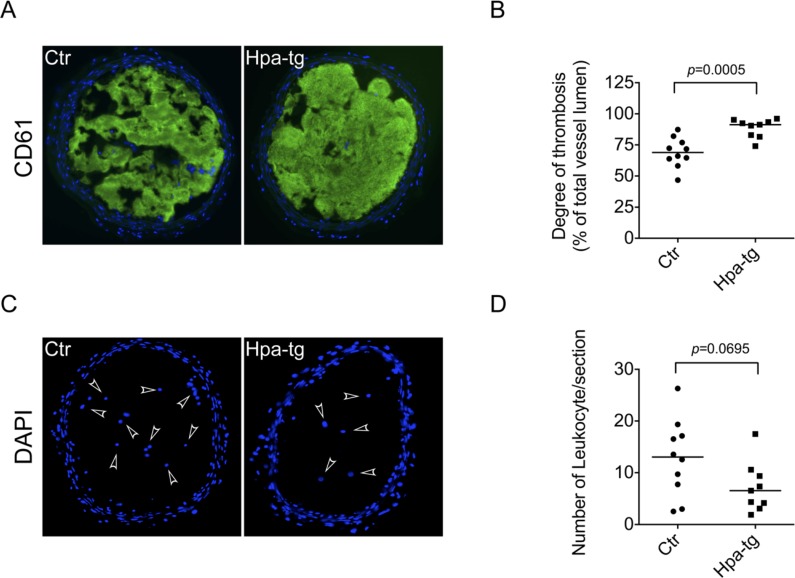
Enhanced thrombosis in carotid arteries of Hpa-tg mice The right carotid of Hpa-tg and Ctr mice were exposed and 2 pieces of filter paper (1×2 mm) saturated with 7.5% FeCl_3_ were applied for 3 min and then removed. The injured site was immediately rinsed with 0.9% NaCl and after an additional 4 min the vessel was dissected, fixed and cryo-sectioned. **A.** Representative sections of Ctr and Hpa-tg carotid tissue show positive CD61 staining in the lumen. **B.** Determination of the CD61 positive signals from 3-4 sections of each mouse (Ctr, *n* = 10; Hpa-tg, *n* = 9). **C.** DAPI staining of the sections (arrowheads indicate leukocytes in the lumen). **D.** Quantification of leukocytes in the sections.

To find out whether heparanase-overexpression affects thrombosis *in vivo*, we applied a carotid injury model for thrombus formation in response to FeCl_3_-induced injury of carotid artery. Considering that artery thrombi are mainly constituted of platelets, degree of thrombosis was assessed by detection of CD61 (integrin β3) positive signals in the carotid section. To overcome the drawback of lacking an ultrasound measurement, we have optimized the experiment by testing several treatment conditions, e.g. FeCl_3_ concentration and duration of treatment before final experiment. We selected a proper treatment window of 7.5% FeCl_3_ for 3 min to visualize the degree of thrombosis. Immunostaining with anti-CD61 antibody revealed that treatment of exposed carotid arteries with FeCl_3_ induced only partial thrombosis in Ctr mice, *vs*. essentially complete clot formation in the carotid of Hpa-tg mice (Figure 5A, Figure S4). Quantification of the fluorescence signals on consecutive sections of the carotid arteries showed a significantly higher intensity of CD61 positive area in the Hpa-tg carotid (Figure 5B), indicating that substantially more platelets were enrolled in response to FeCl_3_ stimulation in Hpa-tg than in Ctr mice. Counting DAPI positive nuclei disclosed marginally fewer leukocytes in the Hpa-tg carotid lumen (Figure 5C, 5D), suggesting that no inflammatory reaction was involved in the hyper-thrombotic activity of Hpa-tg mice in this acute thrombosis model.

## DISCUSSION

Hyper-thrombotic activity is one of the severe complications in cancer patients and platelets are recognized as one of the major contributors to coagulopathy[15]. Previous studies have primarily focused on the functions of platelet heparanase in association with degradation of ECM heparan sulfate (HS), consequently, facilitating extravasation of blood-born leukocytes and tumor cells [8, 16]. Conversely, the implications of platelet heparanase in hemostasis and coagulopathy have largely been overlooked. In the present study, we examined platelet activity with regard to heparanase expression by comparing platelets expressing high *vs*. low levels of heparanase.

Unlike human platelets, wild type C57BL mouse platelets do not express high level of heparanase in comparison to other tissues/cells. However, platelets from transgenic mice overexpressing human heparanase (Hpa-tg)[17] express heparanase at a comparable high level as human platelets (Figure 1A, 1B). Analysis of glycosaminoglycans (GAGs) isolated from platelets revealed that both human and mouse platelets express only CS and no HS or heparin (Figure 1D), in line with previous observations[18], indicating that heparanase expressed in platelets is not destined for cleavage of HS or heparin within the cells.

**Figure 6:**
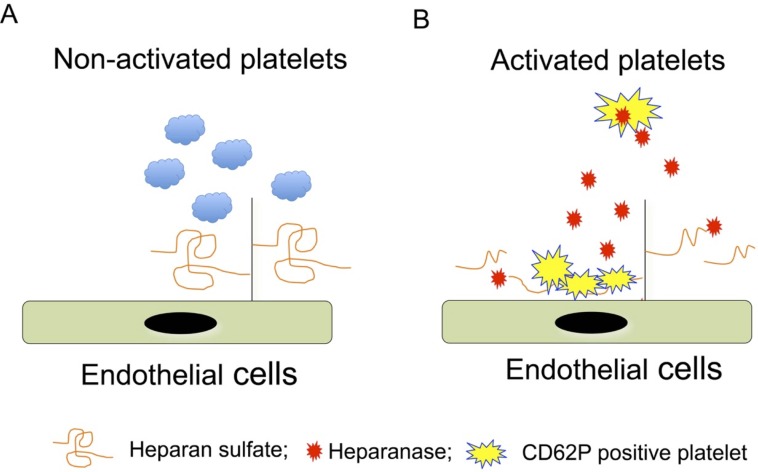
Illustration of the molecular mechanism of heparanase-mediated platelet adhesion **A.** In quiescent platelets CD62P is located in the alpha-granules, and hence does not interact with HS on the endothelial surface. **B.** Activated platelets undergo ‘membrane flipping”, presenting CD62P on the surface. Simultaneously released heparanase cleaves HS on the endothelial surface to expose CD62P binding sites.

Functional implications of heparanase expression in platelets were assessed by adhesion and spreading experiments. *Ex vivo* activated Hpa-tg platelets exhibited higher adhesion and spreading potency in comparison to Ctr platelets. The stronger adhesion activity of Hpa-tg platelets maybe attributed, at least partly, to the associated expression of CD62P, as concomitant upregulated expression of CD62P and heparanase was detected in both human and mouse platelets upon activation. In accordance with previous reports showing interaction of CD62P with HS/heparin [19, 20], our findings plausibly argue for a role of heparanase in CD62P mediated adhesion. The evidence that more wildtype platelets isolated from Ctr mice adhered to HUVEC cells after heparanase treatment than to untreated cells (Figure 2D) strongly supports this argument as illustrated in Figure 6. Heparanase released from activated platelets cleaves HS expressed on endothelial cells, modifying its tertiary structure, thereby facilitating its interaction with CD62P expressed on activated platelets.

As integrins constitute major receptors on platelets, we examined integrins β1 and β3 expression on platelets upon activation with 4 different activators. Unexpectedly, we found that Hpa-tg platelets expressed lower levels of integrin β1 and β3 in comparison to Ctr platelets, with or without stimulations (Figure 4). These data exclude a direct contribution of integrins to the strong adhesion activity of Hpa-tg platelets, in accordance with a previous study showing that β1 integrin serves as an important signaling receptor rather than an adhesion receptor [21]. The reduced level of integrins on Hpa-tg platelets is still sufficient to trigger intracellular signals enabling alpha-granule release of heparanase and CD62P. As CD62P mediated platelet adhesion was found to promote tumor growth[22], the finding of heparanase associated CD62P expression argues for a synergic activity of the molecules released from activated platelet in tumor development.

Since arterial thrombosis, e.g. ischemic stroke and acute myocardial infarction, is characterized by platelet activation and aggregation [23], we applied a carotid artery injury model induced by FeCl_3_. The more rapid and robust development of thrombi in Hpa-tg *vs*. Ctr mice confirms the hyper thrombotic activity of heparanase expression *in vivo* (Figure 5). In consideration of the relative lower expression of integrin β3 (CD61) in Hpa-tg platelets, the strong positive signals in Hpa-tg carotid indicates a robust recruitment and activity of platelets upon injury (activation). Notably, the higher thrombotic activity of Hpa-tg mice likely involves more parameters than platelets, including heparanase expression in the endothelial and smooth muscle cells. However, it is highly conceivable that heparanase expressed in platelets should have made significant contributions to the hyper-thrombotic activity in Hpa-tg mice, as Hpa-tg platelets exhibited a higher adhesion activity onto HUVEC cells. This is in agreement with a previous study showing that thrombosis was markedly advanced on stents incubated with blood taken from Hpa-tg as compared to wild type mice[13].

Thus, it can be proposed that platelet activation stimulates production of heparanase and CD62P. The heparanase released from activated platelets cleaves HS on endothelial cells, thereby exposing binding sites for CD62P that is presented on activated platelets (Figure 6). Since platelets play pivotal roles in cancer metastasis[3, 24], this newly ascribed function of heparanase in platelets provides significant insight for clinical management of metastatic cancer. A rational hypothesis is that heparanase inhibitors could potentially fortify the effects of anti-platelet aggregation drugs. Several heparanase inhibitors are currently under development and clinical testing in cancer patients[25]. Application of such inhibitors may have dual effects on cancer patients with thrombotic complications. These inhibitors are also expected to inhibit platelet-tumor cell aggregation and hence abolish the protective effect of platelets on circulating tumor cells.

## MATERIALS AND METHODS

### Animals

Transgenic mice overexpressing heparanase (Hpa-tg) were generated as described previously[17]. The mice were backcrossed with C57BL mice for more than 10 generations and wild type C57BL were used as control (Ctr). The GFP-C57BL mice were from JAX laboratory (stock 004353, C57BL/6-Tg UBC-GFP). Mice were maintained at the animal facility, Biomedical Center, Uppsala University. The local ethic committee approved all procedures involving animal experiments, and the experiments were conducted in accordance with animal welfare regulations.

### Antibodies and reagents

Rabbit anti-CD61 antibody was purchased from Abcam. Rabbit anti-heparanase antibodies 1453 and 733 were previously described[10]. Rabbit anti-GAPDH, anti-lamp1, anti-CD62P and secondary HRP-conjugated anti-mouse and rabbit IgG were all obtained from Santa Cruz. Anti-integrin antibodies, FITC-conjugated mouse anti-mouse and human CD62P (P-selectin) and isotype IgGs were from BD Pharmingen.

### Platelet preparation and analyses

Whole blood was gently mixed with ACD buffer (6:1), centrifuged at 100xg for 10 min and platelet-rich plasma (PRP) was collected. The PRP was further centrifuged at 800xg for 10 min in the presence of prostacyclin (PGE_1_, 1 μM final concentration; Sigma) and then washed twice with Tyrode's buffer (134 mM NaCl, 12 mM NaHCO_3_, 2.9 mM KCl, 0.34 mM Na_2_HPO_4_, 1 mM MgCl_2_, 10 mM Hepes, pH 7.4). The pelleted platelets were then re-suspended in modified Hepes-Tyrode's buffer (containing 5 mM glucose), counted and used for the following analyses.

#### Western blot analysis

Cells were lysed in buffer (1% NP40, 50 mMTris-HCl, pH 7.4, 150 mM NaCl, and 1 mM EDTA) in the presence of complete protease inhibitor cocktail. The cell lysates were subjected to SDS-PAGE (12%) followed by electro-transfer to PVDF membranes and subsequent blocking in 5% nonfat dry milk dissolved in Tris-buffered saline containing 0.05% Tween. The membranes were probed with primary antibodies against heparanase (1453) and GAPDH, followed by incubation with corresponding secondary antibodies. Signals were developed and visualized using SuperSignal Dura substrate (Thermo scientific) and captured by Chemidoc^TM^ system (Bio-rad). Image J software was used for quantification of band intensity.

#### Immunohistostaining of tissues and cells

Platelets prepared as described below were suspended in Hepes-Tyrode's buffer to a final concentration of 5×10^8^ cells/mL and plated on fibrinogen-coated glass slides, followed by the addition of 10μL of 100μM ADP (activation) or PBS (control). The slides were agitated for 1 min on a shaker at 180 rpm. After equilibration for 3 min, the slides were rinsed twice with PBS, and fixed with 2% paraformaldehyde (PFA) at 4°C for 2 hr. Then, the platelets were treated with 0.2% Triton X-100 for 5 min, rinsed twice, and blocked with 5% BSA in PBS (PBS-BSA) at 37°C for 30 min. The platelets were then incubated with primary antibodies diluted in PBS-BSA at 37°C for 1 hr, followed by incubation with secondary antibodies. After extensive washing in PBS, the coverslips were mounted with VECTASHIELD^®^ Mounting Medium containing DAPI (4,6 diamidino-2-phenylindole) for nuclear counterstaining. Platelets were examined by microscopy as described in the figure legends.

#### Platelet spreading

The platelets (5×10^6^/mL) were incubated at 37°C for 30 min on coverslips pre-coated with fibrinogen (100 μg/mL). After gently rinsing 3 times with Hepes-Tyrode's buffer to remove unbound platelets, remaining adherent platelets were fixed with 1% PFA for 20 min. Images of adhered platelets were acquired by differential interference contrast (DIC) microscopy with a Plan Apo VC 60×/1.40 oil immersion lens of a Nikon ECLIPSE 90i microscope, using Nikon DS-Fi1camera and NIS-Elements AR3.2 software. Images were manually outlined and the degree of platelet spreading was computed off-line by determining the number of pixels within each outline using a Java plugin for the Image J software package.

#### Platelet adhesion

Platelets adhesion to HUVEC cells was performed as previously described [26] with modification. HUVEC cells were cultured on Nunc Lab-Tek chamber slide (Thermo Fisher Scientific) to confluency. Platelets isolated from mice were diluted in Tyrode's buffer containing the stimulators and incubated on the monolayer of HUVEC at 37°C for 30 min. The chamber slides were then rinsed and adherent platelets were visualized by staining with anti-CD61 antibody. Alternatively, fluorescent platelets (from GFP C57BL mice) were incubated on HUVEC cells with or without heparanase pretreatment (2μg/200μl). The images were captured by confocal microscopy using Carl Zeiss LSM 510 METATM instrument. The total platelet coverage area was measured by Image J software.

#### FACS analysis

For mouse samples, equal volume of whole blood (2μL) from Ctr and Hpa-tg (with or without activation with 4 different stimulators) mice were incubated with PE-conjugated anti-mouse integrin β1 (clone HMb1-1) (Biolegend) and FITC-conjugated anti-mouse integrin β3 (CD61) antibody, or anti-active integrin β1 followed with secondary FITC-conjugated goat anti-rat IgG antibody. For human samples, platelets were isolated from whole blood and washed with Hepes-Tyrode's buffer. The platelets were then activated with ADP and incubated with FITC-conjugated anti-human CD62P antibody (BD Pharmingen) and rabbit anti-heparanase antibody 1453. Secondary antibody (goat-anti-rabbit IgG1) labeled with APC was used to detect heparanase. After incubation with the antibodies, samples were washed and analyzed by BD LSR II flow cytometry (BD Biosciences). At least 10,000 platelets per sample were identified based on their forward and side-scatter characteristics and by positive staining with anti-CD61 monoclonal antibody. Isotype-matched antibodies were used as controls for nonspecific binding. The FACS data were processed as mean fluorescence intensity (MFI) using FlowJo (Tree Star Inc., Ashland, USA).

### Isolation and analysis of glycosaminoglycans (GAGs) from cells

Mouse platelets were prepared from 5 mL mixed blood collected from six Hpa-tg and Ctr mice. Human platelets were from mixed blood donated by 3 healthy individuals. After washing with PBS, platelets were lysed in 50 mMTris-HCl/0.5 M NaCl/1% Triton X-100, pH 7.4. Lysates were subjected to protease K (Sigma) digestion (0.8 mg/mL protease in 50 mMTris-HCl/1mM CaCl_2_/1% Triton X-100, pH7.4) for 24 hr at 55°C. The homogenates were boiled (5 min) to inactivate the protease. After centrifugation at 13,000×g for 10 min, the supernatants were recovered and applied onto a 1-mL DEAE-Sephacel column (GE Healthcare Biosciences) pre-equilibrated with 50 mM Tris-HCl/0.1M NaCl, pH7.4. The column was washed with 50 mMNaAc/0.1 M NaCl, pH 4.5 and proteoglycans (PGs) were eluted with 50 mMNaAc/2M NaCl, pH 4.5. The eluted fractions were pooled and desalted on a PD-10 column (GE Healthcare Biosciences), followed by lyophilization to dryness. Aliquots of the samples were treated with buffer, Chondroitinase ABC (0.1 U/sample, Seikagaku, Japan) or Heparinase I/Heparinase III (1 mU/sample, Seikagaku, Japan) for 24 hr at 37°C. After heat inactivation of the enzymes, samples were separated on 15% PAGE and visualized by Alcian blue 8GX (Sigma-Aldrich) staining.

### FeCl_3_-induced carotid artery thrombosis

Adult mice at the age of 12-20 weeks were anesthetized with Avertin (2.5% tribromoethanol, 150-300 mg/kg; Sigma) and secured supine under a microscope. A segment of the right carotid artery was exposed by blunt dissection. Two pieces of filter paper (1×2 mm) saturated with 7.5% FeCl_3_ (ferric chloride) were placed on opposite sides of the carotid artery (one beneath and one above) in contact with the adventitial surface of the vessel for 3 min and then removed. The exposed vessel was washed with sterile saline. Four min after removing the filter paper, animals were euthanized with CO_2_ and the injured carotid artery was carefully dissected and fixed in 4% PFA for analysis. The control treatment with NaCl was performed by the same procedure. The tissues were embedded in O.C.T. and Cryo sections of 10 μm were immunostained with antibodies as indicated in respective figures.

### Real-time PCR quantification of gene expression

Total RNA was extracted from activated and non-activated platelets prepared with the E.Z.N.A total RNA kit I (OMEGA bio-tek). cDNA, prepared with IScriptcDNA Synthesis kit (Bio-Rad), was used for qPCR (SSoFastEvaGreenSupermix, Bio-Rad) quantification. The primers used are: (mouse serglycin sense, 5′-CAGCCAACAGATGAAAGCAA-3′; mouse serglycin antisense, 5′-TGAGGAAAGGGGTAACAGGA-3′; mouse GAPDH sense, 5′-ACTCCACTCACGGCAAATTC-3′; mouse GAPDH antisense, 5′-TCTCCATGGTGGTGAAGACA-3′). Data were normalized against mRNA level of GAPDH from each sample.

### Statistical analysis

Two-tailed unpaired Student's t test was used to determine the significance between population means. The data are presented as mean ± SE.

## SUPPLEMENTARY MATERIAL FIGURES


