# Supplementary material for: Heparanase expression upregulates platelet adhesion activity and thrombogenicity
Source: Oncotarget. 2016 Apr 23;7(26):39486–96. doi: 10.18632/oncotarget.8960 (PMC5129947; doi:10.18632/oncotarget.8960)
Supplement: Supplementary file 1 [file oncotarget-07-39486-s001.pdf]

Heparanase expression upregulates platelet adhesion activity and thrombogenicity

Supplementary Material

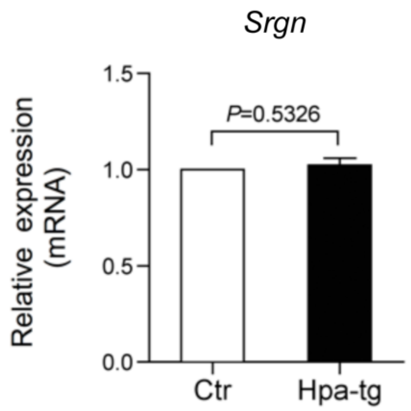

Figure S1. *Quantitative RT-PCR analysis of serglycin (srgn) expression in platelets.* Pooled platelets collected from 3 Hpa-tg and 3 Ctr mice were used and the level of mRNA was quantified by Q-PCR using GAPDH as internal control. The error bars indicate standard deviation from 3 independent experiments performed in triplicates.

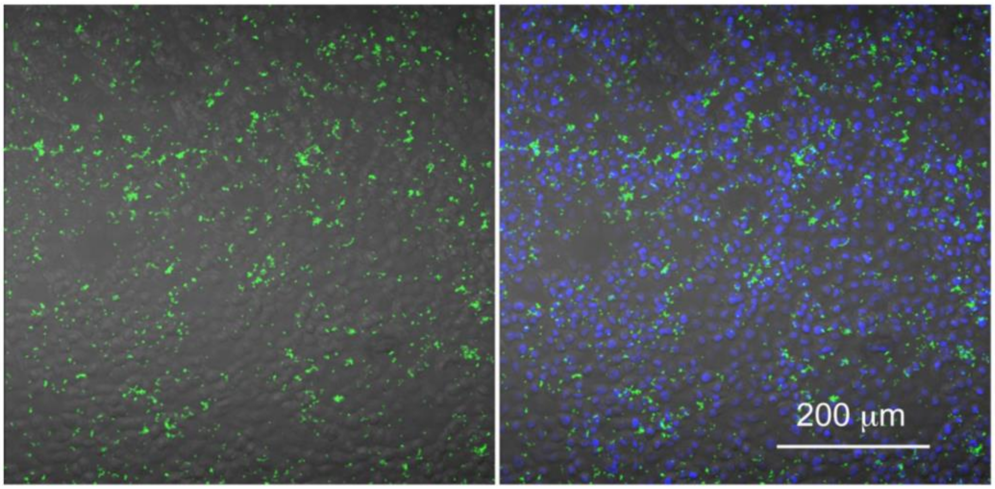

Figure S2. *Image of platelet adhesion to culture HUVEC monolayer.* The platelets were visualized by immunostaining with anti-CD61 antibody (green) and the endothelial cells were visualized by DAPI staining (right panel)

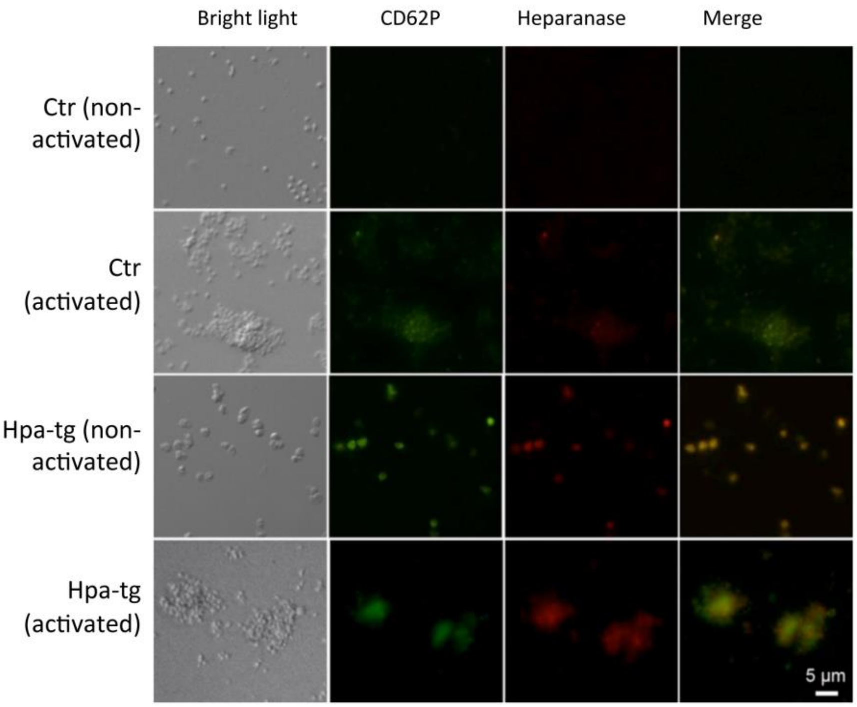

Figure S3. *Examination of heparanase and CD62P expression in mouse platelets upon activation.* Ctr and Hpa-tg platelets suspended in 100 μL Tyrode's buffer (5×10^8 platelets/mL) were plated on fibrinogen-coated coverslips. The platelets were activated by addition of ADP (activated) or PBS (non-activated). After brief agitation and incubation, platelets were fixed and stained with the antibodies indicated in the images of upper panels.

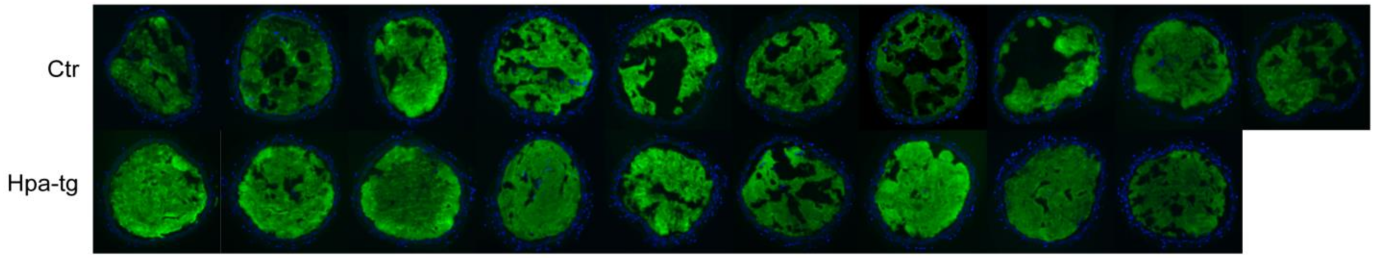

Figure S4. *Arterial thrombosis in a mouse carotid injury model.* Ctr and Hpa-tg mice at the age of 12-20 weeks were used. After exposing the right carotid of anesthetized mice, filter paper of 1x 2 mm saturated with $FeCl_3$ (7.5%) was placed on two sides of the vessel (one beneath and one above) for 3 min and then removed. The exposed vessel was rinsed with saline, and waited for 4 min before dissection. The carotid was fixed in paraformaldehyde and embedded in OCT for cryo-sections. The section of 10 μm was stained with anti-CD61 antibody (Ctr: n=10; Hpa-tg: n=9)
